# Diffuse large B-cell lymphoma: new targets and novel therapies

**DOI:** 10.1038/s41408-021-00456-w

**Published:** 2021-04-05

**Authors:** Bruce D. Cheson, Grzegorz Nowakowski, Gilles Salles

**Affiliations:** 1 Lymphoma Research Foundation, New York, NY USA; 2 Mayo Clinic Foundation, Rochester, MN USA; 3 Lymphoma Service, Memorial Sloan-Kettering Cancer Center, New York, NY USA

**Keywords:** Drug development, Lymphoma

## Abstract

Newer, more effective and non-cytotoxic therapies are an unmet need for patients with diffuse large B-cell lymphoma (DLBCL) and other B-cell malignancies. Recently approved agents include polatuzumab with bendamustine and rituximab, selinexor, and tafasitamab plus lenalidomide. Three CAR-T cell products are currently approved by the FDA, with others in clinical trials. Additional agents in development include bispecific antibodies and antibody drug conjugates. Combinations of targeted therapies should lead to further improvement in the outcome of patients with B-cell malignancies.

## Introduction

The non-Hodgkin lymphomas (NHL) are a diverse group of malignancies about 80% of which are of B-cell origin (B-NHL). B-NHL vary in their presentation, clinical features, prognosis, and response to treatment. The most common histologic subtype is diffuse large B-cell lymphoma (DLBCL) accounting for about a third of cases in the United States, followed by follicular lymphoma which represents about a quarter^1^. The other histologies are much less common. About 60% of DLBCL are cured with regimens such as rituximab, cyclophosphamide, Adriamycin, vincristine, and prednisone (R-CHOP)^2^. However, most patients who relapse following or are refractory to initial therapy succumb to their disease^3^. New drug development over the past decade has appropriately ignored cytotoxic chemotherapy drugs and focused on agents that target the cell surface, internal pathways, and the microenvironment. The chimeric anti-CD20 monoclonal rituximab revolutionized the therapy of B-NHL, prolonging survival in most subtypes. However, resistance eventually develops and other strategies directed at other targets are needed.

Recently, several innovative treatments have been approved by the FDA including the anti-CD79b antibody drug conjugate polatuzumab vedotin (Pola) with bendamustine and rituximab (Pola-BR)^4^; the oral nuclear transport (XPO1) inhibitor selinexor^5^; and, most recently the combination of the anti-CD19 monoclonal antibody tafasitamab with the immunomodulatory agent lenalidomide^6^. In addition are the first two CART-cell products axicabtagene ciloleucel^7^ and tisagenlecleucel^8^. Other drugs in development that target CD20 include the bispecific T-cell engagers (e.g., mosunetuzumab^9^, glofitamab^10^, epcoritamab^11^, and odronextamab^12^). Each of these has exhibited promising early data, including in patients who have progressed following CAR-T therapy^12^.

Polatuzumab vedotin is an antibody-drug conjugate that targets CD79b and delivers the microtubule inhibitor monomethyl auristatin E (MMAE). Polatuzumab vedotin was studied in a phase I, dose escalation trial in 95 patients with NHL or CLL^13^. The primary endpoints were to determine the safety and tolerability, the maximum tolerated dose (MTD), and the recommended phase II dose. In the lymphoma patients, the MTD was 2.4 mg/kg as a single agent and in combination with rituximab. Grade 3-4 adverse events were reported in 58% of patients, most commonly neutropenia (40%), with peripheral sensory neuropathy in 9%. Responses were reported in 54.8% of the NHL patients. The drug was too toxic and had limited activity to be further pursued in CLL. Pola-BR received accelerated approval for R/R DLBCL after two prior regimens (one prior in the European Union) on the basis of a randomized trial comparing Pola-BR with BR, with 40 patients per arm in which Pola-BR achieved a higher complete remission (CR) rate (40.0 vs 17.5%, *p* < .026) and a longer progression-free survival (PFS) (9.5 months vs 3.7 months (*p* = .001) and overall survival (OS) (12.4 vs 4.7 months, *p* = .002). (Table 1) However, Pola-BR was associated with more grade 3-4 neutropenia, anemia, and thrombocytopenia as well as grade 1–2 peripheral neuropathy^4^.

**Table 1:** Tafasitamab monotherapy vs L-mind regimen in NHL.

	Number of patients	Overall response rate	Complete response rate	Median PFS (months)	Median DOR (months)	Reference
Tafasitamab single agent	35 (DLBCL)	26%	6%	2.7	20.1	Jurczak^22^
	34 (FL)	29%	9%	8.8	Not reached	
Lenalidomide single agent	76 (FL)	34.2%	13.2%	4.0	6.6	Nowakowski^25^
Tafasitamab and lenalidomide	81 (DLBCL)	60%	42.5%	12.1	21.7	Salles^6^

*DLBCL* diffuse large B-cell lymphoma, *FL* follicular lymphoma, *PFS* progression-free survival, *DOR* duration of response.

Selinexor is an inhibitor of exportin 1 (XPO1), the major nuclear export protein for a number of tumor suppressor genes and proto-oncogenes. Elevated XPO1 expression inactivates tumor suppressor proteins by mislocalization. Selinexor is a specific inhibitor of XPO1, it reactivates tumor suppressor proteins and blocks proto-oncogene translation, DNA damage repair. The initial phase I trial included 79 patients with NHL, 43 of which had relapse or refractory DLBCL^14^. The most common adverse events included thrombocytopenia in 47%, neutropenia in 32% and fatigue in 11%, with hyponatremia in 10%. In DLBCL, the ORR was 32% with CR in 10% and mDOR of 12.8 months. Activity was also noted in small numbers of patients with follicular lymphoma, CLL, Richter transformation, mantle cell and T-cell lymphomas. The recommended phase 2 dose was 60 mg orally twice weekly. Selinexor received accelerated approval for R/R or transformed DLBCL following two prior regimens on the basis of the SADAL single arm trial in patients with de novo or transformed DLBCL not considered eligible for autologous stem cell transplantation (ASCT) or post-ASCT^5^. These 134 patients had a median age of 67 years, median of two prior regimens, with 53% progressing within a year of their first therapy for DLBCL. This oral agent achieved an ORR of 28% including 13% CRs and with a median duration of response of 9.3 months, but was 23 months for the CRs. At the 60 mg twice weekly dose used in this study, and with intensive anti-emetic support, the drug was well tolerated. The most common toxicity was fatigue in 63%, which was grade 3 or 4 in 15%. Other grade 3–4 toxicities were uncommon. In a subsequent analysis including 134 patients, those <65 years had an ORR of 36.5 vs 24.4% for the older patients, CRs 17.3 and 11%, and median duration of response (DOR) of 9.7 and 9.2 months, respectively.

There have been concerns of a potential favorable selection bias in the SADAL trial in that patients could not have had primary refractory disease, and those with a previous CR or partial remission (PR) to their prior line of therapy were required to wait 60 days from that treatment to initiate selinexor, and 98 days for those with refractory disease^15^. The actual time from progression of disease to selinexor therapy was 1.5 months and 3.3 months, respectively. However, patients in the SADAL study were comparable to typical patients given the patient age, amount of prior therapy. Moreover, 30% had progressed after an autologous stem cell transplant and 72% were refractory to their immediately prior treatment regimen. In addition, the median time from disease progression from the last prior therapy was 59 days in the selinexor responders compared with 52 days in the non-responders, demonstrating that response did not correlate with time since last therapy.

### Targeting CD19

Another potential target is the CD19 antigen. CD19 is a 95 kd, type I, transmembane glycoprotein. Expression of CD19 is specific to B-lymphocytes and follicular dendritic cells on which it is ubiquitous. Expression of CD19 on cells of B-lineage can be through the various stages of differentiation from pre-B cells until plasma cells. CD19 functions as a positive regulator of B-cell receptor (BCR) signaling and is critical for B-cell development, and, in mice the ability to mount an immune response to mitogens, and the production of serum immunoglobulins^16^.

CD19 is present on malignant cells from the majority of patients with NHL, acute lymphoblastic leukemia (ALL) and chronic lymphocytic leukemia (CLL). While CD20 has a higher average density of surface molecules per tumor cell, CD19 expression is more homogenous and is preserved in small CD20-negative tumor subsets and after anti-CD20 targeted therapy. Thus, CD19 serves as an attractive target for lymphoma therapies.

Agents currently in development that target CD19 include tafasitamab, antibody drug conjugates such as loncastuximab tesirine^17^, bispecific T-cell engagers, and CART-cell products including lisocaptagene maraleucel, which was recently FDA approved^18^. Loncastuximab teserine is an antibody-drug conjugate comprised of a humanized anti-CD19 monoclonal antibody conjugated to SG3199, a pyrrolobenzodiazepine dimer toxin. In the phase I^17^, 88 patients with relapsed or refractory NHL and a median of three prior regimens were treated with loncastuximab teserine at doses escalating from 15–200 µg/kg. The most common treatment emergent adverse events (TEAEs) included hematologic abnormalities, fatigue, liver chemistry elevations, nausea, rash, and dyspnea. At doses of >150 µg/kg, the overall response rate was 59.4%, including 40.6% CRs. In the subsequent final report including the dose expansion cohort^19^. one hundred and eighty-three patients were included, with the expansion patients treated at either >120 µg/kg or >150 µg/kg. An MTD was not reached, but >150 µg/kg was chosen as the RP2 dose. The most common TEAEs included febrile neutropenia, fever, pleural effusion, dyspnea, and sepsis. Thirty-five (19.1%) patients experienced TEAEs with a fatal outcome during the study, most commonly (20/35) due to progression of underlying NHL. Six were considered treatment related, all of which were infections. Peripheral edema, pericardial or pleural effusions, and ascites were common and problematic, and elevated liver chemistries were common. For the DLBCL cohort, the ORR was 42.3%, with 23.4% CR; the median DOR was not reached, and the PFS was only 2.8 months.

### Tafasitamab

**Fig. 1:**
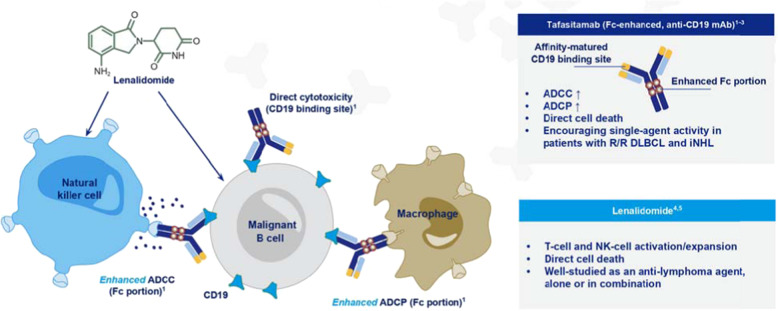
Mechanism of action of tafasitamab and lenalidomide. Tafasitamab has been Fc enhanced to increase antibody-dependent cellular cytotoxicity (ADCC) which augments interactions with natural killer (NK) cells activated and expanded by lenalidomide, antibody-dependent cellular phagocytosis (ADCP) to enhance interactions with macrophages, and direct cell death.

Tafasitamab is an Fc enhanced, humanized anti-CD19 IgG1/IgG2 monoclonal antibody that was engineered to have increased antibody dependent cellular cytotoxicity (ADCC) and antibody dependent cellular phagocytosis (ADCP) with increased cell killing (Fig. 1). The activity of monoclonal antibodies is dependent on interactions with FcγRII, FcγRIII, and FcγRI receptors on effector cells. Natural killer (NK) cells solely express the FcγRIIIa receptor. The majority of monoclonal antibodies in clinical use, such as rituximab, require an interaction between their Fc domain and FcγRIIIa receptor on NK cells for their potency. Tafasitamab has an Fc variant with a two-amino acid substitution at S239D and I332E which increases its affinity to FcγR. The remaining protein sequence of tafasitamab is identical to the IgG1 subclass of monoclonal antibodies in the Fab and hinge regions and identical to the IgG2 subclass of monoclonal antibodies in the CH2 and CH3 domains.

Tafasitamab has demonstrated additive or synergistic activity in vitro with a number of chemotherapy agents including bendamustine, fludarabine, rituximab, and ofatumumab.

#### Phase I study

In an initial phase I trial, tafasitamab was administered to 27 patients with CLL at doses escalating from 0.3–12 mg/kg, for nine infusions on days 1, 4, 8, 15, and 22 of cycle 1 and days 1, 8, 15, and 22 of cycle 2. Following the two cycles, patients without progressive disease were allowed to continue tafasitamab every 28 days for four additional infusions^20^. After demonstrating excellent tolerability, an expansion cohort was treated at 12 mg/kg. All patients experienced at least one TEAE, 24 of which were thought possibly related to the study drug. Grade 3 or 4 toxicities included neutropenia (11%) (including a DLT of grade 4 neutropenia), thrombocytopenia (7.4%), elevated aspartate aminotransferase (3.7%), and tumor lysis syndrome (3.7%). Clinical responses occurred in 66.7% with 29.8% responding by CT criteria.

Pharmacokinetic studies were performed on 25 patients and showed a two compartment model. The clearance and volume of distribution were similar to other full length monoclonal antibodies with a maximum concentration (Cmax) increasing in a somewhat less than a dose-proportional manner supporting that distribution was limited to the systemic circulation. The clearance and half-life were not dose dependent. There was a trend of accumulation in concentration with each infusion achieving a plateau state by infusion 9. In the studied range from 3–12 mg, the drug half-life averaged 13.5 days allowing dosing intervals of 1 to 3 weeks.

#### Phase II studies in CLL

The phase I data supported the COSMOS study of tafasitamab in patients with CLL and small lymphocytic lymphoma (SLL)—who had progressed following a Bruton tyrosine kinase (BTK) inhibitor^21^. Therapy involved treatment with tafasitamab and either the PI3k inhibitor idelalisib (Cohort A) or venetoclax (Cohort B). Tafasitamab (12 mg/kg) was administered in 28-day cycles. In cycles 1–3 tafasitamab was infused on day 1, day 8, day 15, and day 22 with an additional loading dose on day 4 of cycle 1. In subsequent cycles, tafasitamab was administered on days 1 and 15 for cycle 4–6 monthly cycles, and only on day 1 from cycle 7 to 24 or until disease progression or intolerance. Cohort A enrolled 11 patients who received a median of five prior lines of therapy, more than half of which had a complex karyotype or del17p/TP53. The most common grade 3 or higher TEAEs were neutropenia (46%), anemia (27%), thrombocytopenia (27%), pneumonia (27%), and elevated transaminases (18%). Fatal heart failure occurred in one patient. The ORR in Cohort A was 90.9% with 9.1% CRs. Two of eight patients who were assessed for minimal residual disease (MRD) status were undetectable in the peripheral blood (PB), and one of three in the bone marrow (BM). Cohort B included 13 patients who had received a median of three prior lines of treatment, more than 90% of which had a complex karyotype and 69% unmutated IGHV status. The most common grade 3 or higher TEAEs were neutropenia (46%), hypophosphatemia (31%), and infusion related reactions (IRR) (15%). The ORR was 76.9% with 23.1% CRs. Of the seven patients assessed for MRD status, six became undetectable in the PB, and two of four patients in the BM.

#### Phase II studies in non-hodgkin lymphoma

Tafasitamab has been studied most extensively in NHL. The first phase II trial involved 92 patients with various histologies of NHL including 35 DLBCL, 34 FL, 12 MCL, and 11 other indolent non-Hodgkin lymphoma (iNHL)^22^. Tafasitamab was administered at a dose of 12 mg/kg on day 1, 8, 15, and 22 of cycles 1 and 2, then every 2 weeks onward, and patients who achieved at least a PR were eligible to continue treatment until intolerance or progression of disease. The ORR in relapsed/refractory DLBCL, FL, and other iNHL was 26, 29, and 27%, respectively (Table 1). Of six patients with mantle cell lymphoma, there were no responses and this cohort was terminated. The most commonly reported TEAEs were IRR and neutropenia, both occurring in 12% of patients. Respiratory tract infection and headache were reported in 11% each. The most common grade 3 or higher adverse event was neutropenia (9%) which mainly occurred during the first two cycles, with recovery within 1 week (Table 3). The incidence of serious adverse events (SAEs) in the study was 30%; however, in only four patients (4%) was there a suspected relationship to tafasitamab. Two SAEs occurred in patients with DLBCL (febrile neutropenia and genital herpes) and two with FL (dyspnea and myelodysplastic syndrome). There were no treatment related deaths.

#### Combination regimens

Supported by the modest single agent response rate of tafasitamab in DLBCL, combinations with other agents were developed. To date, the most promising of partners has been the immunomodulatory drug, lenalidomide. The rationale is that the antibody augments cellular immunity against the lymphoma cell target. The Fc enhanced portion of tafasitamab has increased affinity to Fcγ receptors such as FcγRIIIa. These FcγRIIIa receptors, present on immune effector cells such as NK cells, mediate the ADCC response. Lower PB NK cell count has been associated with poorer clinical outcomes in patients with DLBCL supporting an essential role of NK cells in ADCC. In addition, the antibody induces ADCP, augmenting macrophage cell killing. Lenalidomide has been well studied in lymphoma both as a single agent and in combinations. In vitro studies demonstrated that NK-cell mediated ADCC with tafasitamab was further enhanced by lenalidomide.

**Fig. 2:**
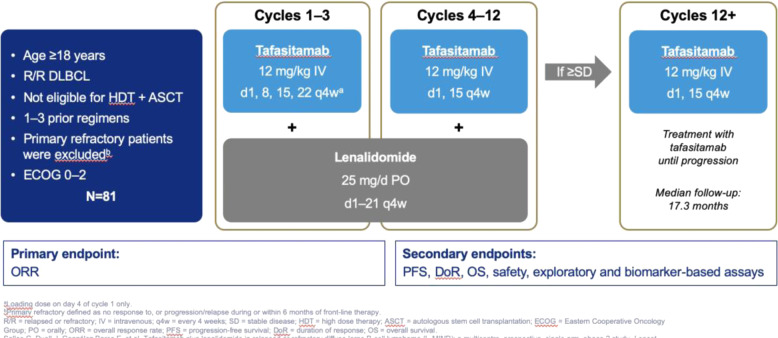
Schema of the L-MIND regimen. Patients receive the combination of tafasitamab and lenalidomide for 12 cycles, and those with at least stable disease can continue on single agent tafasitamab until disease progression or intolerance.

These observations led to the L-MIND study, a phase II trial of the combination of lenalidomide and tafasitamab (Fig. 2)^6^. Eligibility required age of at least 18 years with relapsed or refractory (but not primary refractory) DLBCL and ECOG performance status of 0–2. Patients could not be considered eligible for ASCT by their primary physician on the basis of age or the presence of comorbidities. A small subset entered the trial after refusal of ASCT. Tafasitamab was administered at a dose of 12 mg/kg iv on days 1, 4, 8, 15, and 22 for cycle 1, days 1, 8, 15, and 22 for cycles 2–3, and days 1 and 15 for subsequent cycles. Lenalidomide was dosed at 25 mg a day, day 1–21 of each 28-day cycle, higher than the 20 mg dose used in the rituximab plus lenalidomide regimen. Patients with stable disease or better after 12 monthly cycles were eligible to continue tafasitamab as a single agent until disease progression or intolerance. The primary endpoint was ORR, with secondary endpoints including PFS, DOR, safety and a number of exploratory biomarker studies.

There were 81 patients accrued to the study. Patient characteristics included a median age of 72 (41–86) years. Fifty-four percent were male and 75% had stage III or IV disease. IPI 0–2 were in 49% and an elevated LDH was noted in 56%. These patients had limited adverse features. Whereas they had received a median of two prior lines of therapy, 49% only had one prior and 43% two prior therapies. Only 15 patients (18%) were considered to have primary refractory disease. Such patients were not initially considered eligible for the study; however, the definition of “primary refractory” changed during the conduct of the trial from progression within 3 months to progression within 6 months and the 15 patients had been within the 3–6 month window. In addition, 44% were considered refractory to their prior line of therapy. Eleven percent underwent a prior stem cell transplant. Cell of origin was germinal center B-cell (GCB) in 46%, non-GCB in 25%, and unknown in 30%. Of note is that CART-cell patients are also a selected population, and the 40% long-term duration of response is not dissimilar from that reported with either autologous stem cell transplant or L-MIND in transplant ineligible patients.

**Fig. 3:**
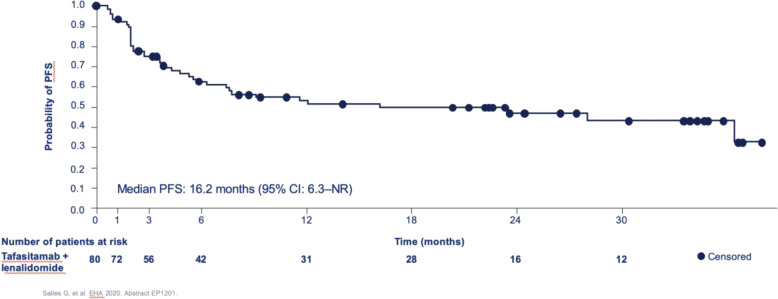
Progression-free survival in the L-MIND Study (courtesy of Morphosys). The median PFS is 16.2 months (95% CI: 6.3-NR).

**Fig. 4:**
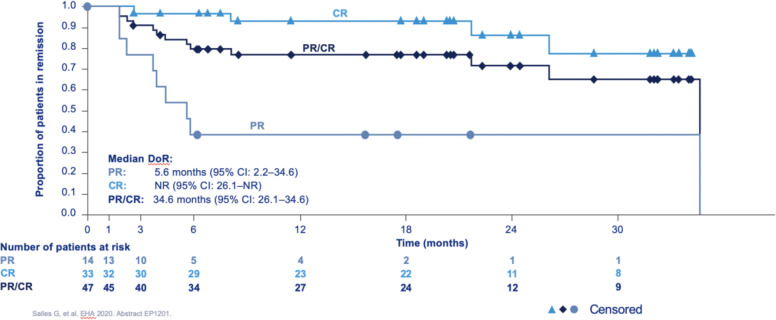
Duration of response in the L-MIND study (courtesy of Morphosys). DOR correlates with response in DLBCL with NR in those with a CR and only 5.6 months for those with a PR.

In updated results, as of November 2019, the ORR was 58.8% with 41.3% CRs^23^. The median PFS was 16.2 months with a median survival of 31.6 months (Fig. 3). With at least 24 months of follow-up, the median DOR was 34.6 months (Fig. 4). Of note was that the median DOR was not reached for the CRs, but was 5.6 months for the PRs, confirming the importance of achieving a CR. At the time of this publication there were 22 patients still on therapy.

This regimen was well-tolerated with the major adverse effects being neutropenia: grade 3 and 4 in 27 and 21%, respectively, but without infections. Grade 3 thrombocytopenia was reported in 17%. Non-hematologic toxicities included rash (grade 1–2 in 27%, grade 3 in 9%); grade 1–2 diarrhea in 32%, but grade 3 in only 1%; other toxicities occurring in at least 20% of patients included asthenia (21%), cough (21%), peripheral edema (22%), fever (20%), and decreased appetite (20%); however, grade 3 or worse toxicities were uncommon (Table 2). Treatment related SAE occurred in 18.5% of patients with 10% experiencing infections and 5% with febrile neutropenia. There were four treatment related deaths (sudden death, respiratory failure, cerebrovascular accident, progressive multifocal leukoencephalopathy), none thought to be related to study drugs. The marked reduction in toxicity with the prolonged tafasitamab as a single agent suggests that the toxicity during induction was primarily the result of lenalidomide. Despite the 25 mg starting dose of lenalidomide, 80% of patients were able to remain on at least 20 mg. About half the patients received growth factor support at the discretion of the treating physician in order to maintain dose intensity, which is considered important for the efficacy of the regimen.

**Table 2:** Adverse events of grade 3 or higher encountered in NHL patients in phase II studies.

Events	Tafasitamab single agent^a^	Tafasitamab and lenalidomide^b^
Neutropenia	9%	48%
Thrombocytopenia	4%	17%
Anemia	3%	7%
Febrile neutropenia	-	2%
Dyspnea	4%	-
Pneumonia	3%	6%
Fatigue	2%	2%
Hypokalemia	2%	1%
Rashes	-	9%
Hypokalemia	-	6%
Urinary tract infection	-	5%
Hypertension	-	4%
Pulmonary embolism and deep vein thrombosis	-	4%
Atrial fibrillation	-	3%
Upper respiratory tract infection	-	2%
Congestive heart failure	-	2%
Increased transaminases	-	2%
Renal failure	-	2%
Back pain	-	2%
Asthenia	-	2%

^a^Encountered in two or more patients.

^b^In at least 2% of the patients.

In the L-MIND study, grade 3 and 4 neutropenia were reported in 27 and 21%, respectively; however, febrile neutropenia in only 10 and 2%, respectively. Neutropenia was managed by granulocyte colony-stimulating factor in 44% of patients, including 81% of those with grade 3 or 4 neutropenia. The majority of these cases returned to normal neutrophil count within a week.

Management of neutropenia with L-MIND is similar to the published recommendations for ritiuximab plus lenalidamide (R^2^)^24^. When neutrophils fall below 1000/µl for at least 7 days or below 1000/µl in the setting of a fever >38.5 °C, or <500/µl, lenalidomide should be interrupted and blood counts checked weekly. When neutrophils return to >1000/µl, resume at a dose 5 mg/day less than the starting dose. The routine prophylactic use of a growth factor is not recommended; however, such an agent should be considered in the setting of grade 3 or 4 neutropenia lasting >7 days or in patients with recurrent neutropenia to maintain the lenalidomide dose.

The efficacy of L-MIND appears to be superior to the 29% ORR previously reported with tafasitamab monotherapy (Table 1). However, at the request of the FDA, the RE-MIND study was designed as a non-interventional data comparison of patients treated with L-MIND with those treated with single agent lenalidomide who were matched for nine relevant clinical and laboratory features; age, stage, refractoriness to last line of therapy, number of prior therapies, history of prior refractoriness, prior ASCT, neutropenia, anemia, and elevated LDH^25^. The primary endpoint was best ORR determined by the investigator. There were 490 patients identified with lenalidomide therapy alone of which 76 matched with 76 of those patients from the L-MIND study. The ORR and CR rates with L-MIND were 67.1% and 39.5%, respectively, and for lenalidomide monotherapy 34.2% and 13.2%, respectively (*p* < 0.0001). The PFS was 12.1 months vs 4 months (*p* = .0002; HR 95% CI 0.307–0.698) and overall survival was also significantly longer at not reached vs 9.4 months for the L-MIND regimen vs lenalidomide monotherapy, respectively (*p* = .0026) (HR 95%CI, 0.317–0.785) (Table 1). RE-MIND2 is being planned in which the data with tafasitamab and lenalidomide will be compared in a similar fashion with patients treated with a variety of other options.

Based on these data, L-MIND was granted accelerated approval by the FDA on August 3, 2020.

Whether L-MIND is superior to R^2^ in DLBCL is unknown as there have been no direct comparisons. Wang et al. reported on 32 patients with relapsed or refractory DLBCL, transformed or grade 3 follicular lymphoma at a median age of 65 years and with a median of three prior lines of therapy^26^. The ORR was 33% with 22% CR, a median PFS of 2.8 months and a median OS of 10.2 months. For the DLBCL subset (*n* = 32), the ORR was 28% including 22% CRs. Zinzani et al.^27^ reported a series of 23 elderly patients with relapsed or refractory disease. The ORR was 35% with 30% CR, a 1-year disease-free survival rate of 34.8% and an 18 month OS of 55.1%. Despite differences in patients, the results with L-MIND appear superior to either of these studies.

Other tafasitamab combinations are in development. The B-MIND study is the confirmatory trial for L-MIND and it is an ongoing comparison between bendamustine and rituximab vs bendamustine tafasitamab in relapsed and refractory DLBCL. FIRST-MIND is a phase 1b study exploring the relative tolerability of R-CHOP-tafasitamab vs R-CHOP-tafasitabmab+lenalidomide. The more favorable regimen will likely be compared with R-CHOP in a phase III trial in previously untreated DLBCL.

## Toxicities of tafasitamab

Tafasitamab as a single agent and in combination with lenalidomide is generally well tolerated. The most common adverse events are listed in Table 2 which suggests that most of the toxicities in the L-MIND study were related to the lenalidomide.

## Tafasitamab administration and dose modifications

Patient treated with tafasitamab should receive premedication with acetaminophen, histamine H_2_ receptor antagonists and/or glucocorticoids. Premedication should be administered 30 min to 2 h prior to starting tafasitamab administration. Subsequent premedication is optional for patients who do not experience an infusion-related reaction during the first three infusions. However, patients who continue to experience infusion-related reactions should receive premedication before each subsequent infusion. The dose of tafasitamab is 12 mg/kg intravenously days 1, 4, 8, 15, and 22 of cycle 1; days 1, 8, 15, and 22 of cycles 2 and 3; days 1 and 15 of cycles 4–12 and for subsequent cycles for patients whose response is at least stable disease until progression of disease or intolerance. The first infusion should be administered at a rate of 70 mL/h for the first 30 min, then, the rate can be increased, in the absence of an infusion reaction, so that the infusion is administered within 1.5 to 2.5 h. All subsequent infusions can be delivered within 1.5 to 2 h.

Dose modification recommendations for hematologic toxicity are outlined in Table 3.

**Table 3:** Dose modifications for tafasitamab and lenalidomide hematologic toxicities.

Event	Dose modification
Platelet count ≤50,000/µl	Hold tafasitamab and lenalidomide and monitor counts weekly until platelet count is ≥50,000/µl
Neutrophil count ≤1000/µl for at least 7 days or febrile neutropenia or neutrophil count ≤500/µl	Hold tafasitamab and lenalidomide and monitor counts weekly until neutrophil count is ≥1000/µl

## Future directions

Several active targeted therapies have been approved by the FDA for patients with relapsed or refractory DLBCL, with numerous others in development. The L-MIND regimen is the first therapy approved for second and subsequent lines of therapy patients not considered candidates for ASCT. As noted above, studies are ongoing and planned to determine the role of tafasitamab as part of initial therapy for patients with this histology. Patients with DLBCL are often elderly with comorbidities. Combining L-MIND with mini-CHOP or comparing L-MIND + rituximab with mini-CHOP are questions worthy of study in such a population.

Patients with relapsed or refractory disease have progressed after anti-CD20 therapy. Thus, switching to CD19-direted therapy may improve patient outcome. Incorporating tafasitamab into a pre-ASCT regimen is a question worth study.

Concerns have been expressed that using an anti-CD19 agent may compromise subsequent CART-cell therapy. However, levels of CD19 are maintained following tafasitamab therapy and anecdotes suggest successful cellular therapy in patients previously treated with L-MIND. CART-cell therapy is an extremely promising therapy for DLBCL, MCL, and FL. However, the majority of patients do not benefit and face disease progression. Tafasitamab-based therapy should be tested in this setting, except in those patients whose cells no longer express CD19.

Based on their mechanism of action, the activity of targeted agents as anti-CD47 antibodies, check point inhibitors, or bispecific T-cell engagers, might be augmented by L-MIND.

As CD19 is expressed on virtually all B-cell malignancies, a logical step is to test tafasitamab alone or with lenalidomide in other histologies, notably FL and marginal zone lymphoma (MZL). Since early studies did not suggest activity in MCL, further pursuit is of lower priority. The rituximab/lenalidomide (R^2^) regimen has been shown to be effective in the relapsed/refractory and front-line settings. Whether substituting tafasitamab for rituximab or adding the two antibodies are two approaches that should be considered. Combinations of monoclonal antibodies in lymphoid malignancies has been shown to be effective in earlier studies. Such regimens could not only be studied in the relapsed refractory setting, as in the AUGMENT study, but also in the front-line setting. Other combinations with PI3k or BTK inhibitors may be worthy of testing in appropriate histologies. Future directions include combinations with other agents or substitution for rituximab in patients previously treated with the anti-CD20 monoclonal antibody.

Tafasitamab may also serve as a backbone for the development of novel strategies for patients with other histologies of NHL.

Having an increasing number of active therapies for DLBCL raises a number of issues. First is how best to sequence them. At present, L-MIND is the only therapy approved for patients failing a single prior systemic regimen. For patients progressing after this regimen and not suitable for CART therapy, the decision is between selinexor and Pola-BR (Table 4). The former has an advantage for ease of administration and a favorable safety profile. Whereas the latter achieves a higher CR rate and longer PFS, the DOR rates were comparable. Moreover, an advantage for selinexor is its ease of incorporation into combination strategies, many of which are currently in development.

**Table 4:** Response and outcomes results for regimens for the treatment of relapsed and refractory DLBCL.

Efficacy metric	Selinexor (*n* = 134)	Pola-BR (*n* = 40)	L-MIND (*n* = 81)	R-GemOX (*n* = 49)	R-GDP (*n* = 52)	R^2^ (*n* = 32)
ORR, %	29	45	57.5	61	63	28
CR, %	13	40	40	44	31	22
mDOR (mo)	9.3	12.6	34.6	10	NR	6
mPFS (mo)	2.6	9.5	12.1	5	3 yr–31%	2.8
mOS (mo)	9.0	12.4	31.6	11	3 yr–66%	10.2

Thus, with the increasing availability and approval of novel therapies for DLBCL, recommendations for an optimal paradigm is challenging and dynamic. At present, the L-MIND regimen is the only FDA approved second-line therapy, although polatuzumab + BR has recently received compendium status. CART therapy is currently approved in the third line; however, three ongoing trials are evaluating this therapy in the second line (axicaptagene ciloleucel, tisagenlecleucel, and lisocaptagene maroleucel), and one in front-line, high-risk patients^28^. Thus, patients who are relapsed/refractory to R-CHOP are distinguished by their eligibility for stem cell transplantation. For those who are not, L-MIND is the approved option. Therapies for the third line include CAR T for those who are eligible and who have access to the procedure, or selinexor and poltazumab-BR; the oral administration and safety profile of the former are in its favor; however, the CR rate and PFS support the latter. Should loncastuximab teserine receive approval, its position in the paradigm will be determined by the line of treatment for which is indicated.

How best to move these regimens earlier in the course of the disease where they are likely to be more effective is an important consideration. The POLARIX trial comparing R-CHOP with R-CHP + polatuzumab has been completed. If the results favor the investigational arm, L-MIND would remain as second line followed by selinexor or CART. If the upcoming tafasitamab-R^2^CHOP vs R-CHOP front-line study is positive, it would become a preferred option for initial therapy. If both are positive, treating physicians would have to decide between them on the basis of relative safety and efficacy. Tafasitamab use in later lines of therapy would then be limited to patients who had not already received that agent. For the other patients, Pola-BR in patients naïve to those drugs would be a consideration as it has compendium acceptance in second line. Should CAR T be approved in the second line, the selection of therapy among CART T, L-MIND, and Pola-BR would be determined by eligibility criteria, availability, toxicity concerns, financial considerations, and other factors. ADCs, bispecifics, and CART approaches are rapidly moving earlier in the course of the disease. Indeed, mosunetuzumab has been evaluated as the initial line of treatment in older patients with DLBCL or those with comorbidities with impressive results^9^. With the impending availability of safer and effective CART products, such as lisocaptagene maraleucel^18^ more patients may be referred for that therapy.

The greatest progress will result from the development of rational combinations with improved efficacy and a favorable safety profile. While the abovementioned trials are underway, identification of biomarkers are essential to help select individual patients for optimal therapies. Such novel chemo-free approaches will certainly lead to improved outcomes for patients with DLBCL and other histologies of B-cell lymphomas.
